# Rare-Earth Metal-Based Materials for Hydrogen Storage: Progress, Challenges, and Future Perspectives

**DOI:** 10.3390/nano14201671

**Published:** 2024-10-18

**Authors:** Yaohui Xu, Xi Yang, Yuting Li, Yu Zhao, Xing Shu, Guoying Zhang, Tingna Yang, Yitao Liu, Pingkeng Wu, Zhao Ding

**Affiliations:** 1 Laboratory for Functional Materials, School of New Energy Materials and Chemistry, Leshan Normal University, Leshan 614000, China; 2 Leshan West Silicon Materials Photovoltaic New Energy Industry Technology Research Institute, Leshan 614000, China; 3 Yunnan Energy Research Institute Co., Ltd., Kunming 650299, China; 4 College of Materials Science and Engineering, National Engineering Research Center for Magnesium Alloys, National Innovation Center for Industry-Education Integration of Energy Storage Technology, Chongqing University, Chongqing 400044, China; 5 Department of Electrical and Computer Engineering, Illinois Institute of Technology, Chicago, IL 60616, USA; 6 Department of Chemical Engineering, Illinois Institute of Technology, Chicago, IL 60616, USA

**Keywords:** rare-earth metals, hydrogen storage, nanostructuring, surface modification, catalytic doping, thermodynamics, kinetics

## Abstract

Rare-earth-metal-based materials have emerged as frontrunners in the quest for high-performance hydrogen storage solutions, offering a paradigm shift in clean energy technologies. This comprehensive review delves into the cutting-edge advancements, challenges, and future prospects of these materials, providing a roadmap for their development and implementation. By elucidating the fundamental principles, synthesis methods, characterization techniques, and performance enhancement strategies, we unveil the immense potential of rare-earth metals in revolutionizing hydrogen storage. The unique electronic structure and hydrogen affinity of these elements enable diverse storage mechanisms, including chemisorption, physisorption, and hydride formation. Through rational design, nanostructuring, surface modification, and catalytic doping, the hydrogen storage capacity, kinetics, and thermodynamics of rare-earth-metal-based materials can be significantly enhanced. However, challenges such as cost, scalability, and long-term stability need to be addressed for their widespread adoption. This review not only presents a critical analysis of the state-of-the-art but also highlights the opportunities for multidisciplinary research and innovation. By harnessing the synergies between materials science, nanotechnology, and computational modeling, rare-earth-metal-based hydrogen storage materials are poised to accelerate the transition towards a sustainable hydrogen economy, ushering in a new era of clean energy solutions.

## 1. Introduction

**Figure 1:**
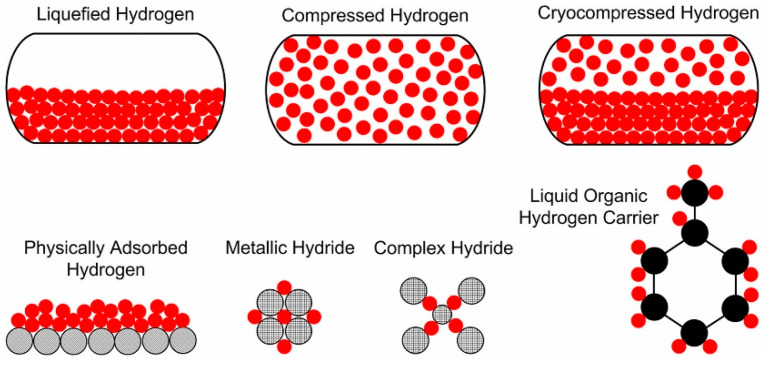
Concept of hydrogen storage methods (Red is H atom, Black is carbon) [17].

The rise in global temperatures and the gradual depletion of fossil fuel resources have made the global demand for clean and renewable energy increasingly urgent [1,2,3,4]. Reducing carbon emissions, particularly carbon dioxide emissions, has become a common goal for governments and organizations worldwide [5,6,7,8,9]. Among the various alternative energy sources, hydrogen stands out due to its high energy density and zero carbon emissions upon combustion, making it a crucial component of future energy systems [10,11,12]. When hydrogen burns, it only produces water, releasing no harmful substances, which positions it as one of the most environmentally friendly energy sources [13,14]. However, the widespread adoption of hydrogen energy still faces numerous challenges, particularly in the efficient, safe, and practical storage of hydrogen [15,16]. As illustrated in Figure 1, hydrogen storage technologies can be broadly categorized into three main types: gaseous, liquid, and solid-state [17]. Gaseous hydrogen storage primarily involves high-pressure gas storage in specialized containers. While this method is straightforward, it suffers from low hydrogen mass density and poses safety risks due to the potential for leakage and explosion [18]. Liquid hydrogen storage is divided into cryogenic and organic liquid storage. Cryogenic storage requires maintaining extremely low temperatures, leading to high energy consumption and increased complexity and cost in storage and transportation [19,20]. Organic liquid storage, while offering higher densities, often involves toxic and hazardous substances prone to leakage [21,22,23]. In response to the challenges presented by gaseous and liquid storage methods, solid-state hydrogen storage has emerged as a promising alternative [24,25,26]. This category includes several subtypes: (1) physical adsorption (physisorption) using high-surface-area materials like activated carbons, zeolites, or metal–organic frameworks (MOFs); (2) metal hydrides, which form stable metal–hydrogen compounds; (3) complex hydrides, typically lightweight compounds containing alkali or alkaline earth metals; (4) chemical hydrogen storage materials, which release hydrogen through chemical reactions. These solid-state methods offer the potential for higher hydrogen storage densities and enhanced safety compared to gaseous and liquid storage [27,28,29]. Each subtype presents unique advantages and challenges in terms of storage capacity, kinetics, and operating conditions, contributing to the diverse landscape of hydrogen storage technologies and driving ongoing research in the field.

In recent years, researchers exploring various new hydrogen storage materials have discovered that rare-earth metals exhibit tremendous potential in this field due to their unique physical and chemical properties [30,31,32]. Particularly, the lanthanides (elements with atomic numbers 57–71) are considered highly promising for hydrogen storage [33,34,35,36]. These metals have a high affinity for hydrogen, forming stable hydrides that significantly increase hydrogen storage density, exhibiting excellent reversibility and multiple hydrogen storage mechanisms [21,37,38]. Moreover, rare-earth metals and their compounds, when used as catalysts or alloying components in hydrogen storage materials, can enhance storage capacity and release rates, further expanding their application prospects. Despite the low natural abundance and high extraction costs of rare-earth metals, they are currently more commonly used as key components in alloys or catalysts to optimize existing hydrogen storage technologies [39,40]. With advancements in technology and the continued growth in demand for clean energy, the role of rare-earth metals in future energy systems is expected to expand, contributing significantly to achieving clean and efficient energy transitions.

This paper aims to elucidate the critical role of rare-earth metals in advancing hydrogen storage technologies and to highlight their potential to transform the energy landscape. By addressing both fundamental and applied aspects of hydrogen storage materials, this review seeks to provide valuable resources for researchers and practitioners striving to harness the full potential of hydrogen as a clean and renewable energy source.

## 2. Hydrogen Storage Mechanisms of Rare-Earth Metals in Alloys

The hydrogen storage mechanism in alloys primarily involves adsorption, dissociation, diffusion, and hydride formation. The unique properties of rare-earth metals, including their valence states, crystal structures, and electronic configurations, play crucial roles in these processes [41,42]. The localized 4f orbitals in rare-earth metals significantly influence their hydrogen storage performance. Factors such as atomic size, surface catalytic activity, and conduction band properties formed by outer electrons directly affect their storage capabilities [43]. For instance, the structural and performance differences between divalent EuH_2_ and trivalent EuH_3_ demonstrate how varying valence states impact hydride formation and stability. Most rare-earth metals maintain a +3 oxidation state in their hydride phase, leading to distinct hydrogen storage behaviors [44,45].

During the interaction between rare-earth metals and hydrogen, the high affinity for hydrogen results in the absorption of hydrogen molecules on the metal surface [46]. These hydrogen atoms are then adsorbed into the interstitial positions of the metal lattice, forming new hydride phases with specific stoichiometry and crystal structures. The introduction of rare-earth elements can significantly enhance the dissociation and diffusion rates of H_2_ in these processes, thereby improving overall hydrogen storage performance. However, the fundamental mechanisms and dynamic migration pathways of hydrogen in these systems remain insufficiently studied [47]. A deeper understanding of these processes is critical for comprehending and optimizing the hydrogen storage behavior of rare-earth-based alloys [48,49]. Further research in this area could lead to significant advancements in hydrogen storage technology.

Advanced techniques are crucial for analyzing and optimizing rare-earth-metal-based hydrogen storage materials. X-ray diffraction (XRD) identifies hydride phases, monitors phase transitions, and evaluates structural stability, with in situ XRD offering real-time insights. Scanning electron microscopy (SEM) and transmission electron microscopy (TEM) reveal particle morphology and atomic-scale structures, providing detailed internal images. Hydrogen absorption/desorption isotherms assess storage capacity and thermodynamic properties, elucidating storage mechanisms. Temperature-programmed desorption (TPD) and temperature-programmed reduction (TPR) examine desorption behavior and reducibility, highlighting different binding sites and material stability.

**Figure 2:**
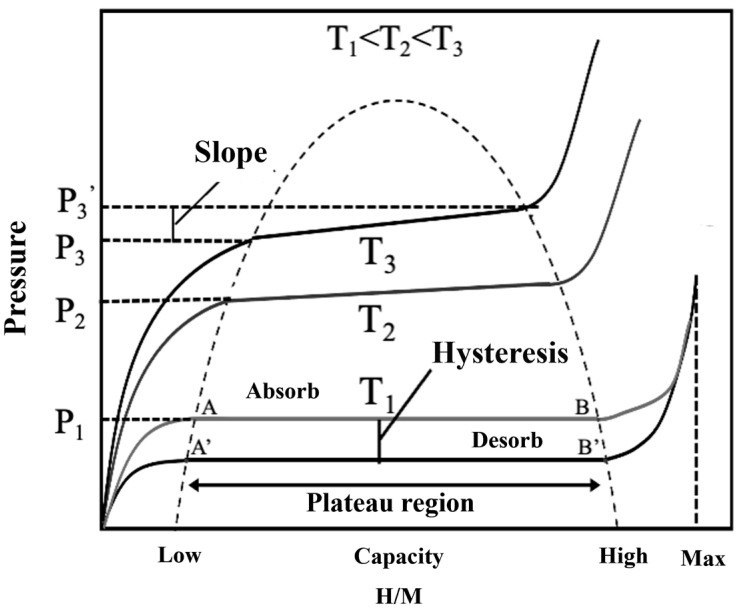
Depicts the pressure–composition isotherms indicating the amount of hydrogen absorbed and released at different pressures and temperatures. The shape of the curve provides insights into storage mechanisms, such as chemisorption or physisorption, and the reversibility of the storage process.

The pressure–composition–temperature (P-C-T) curve includes crucial data for analyzing the hydrogen storage performance of materials. As shown in Figure 2, the plateau regions AB/A’B’ of the P-C-T curve represent the reversible transformation between metal and metal hydride resulting from chemical reactions induced by hydrogen dissolution/desorption. Comparing the width of this plateau helps analyze the reversibility of the hydrogen absorption/desorption reactions—the wider the plateau, the more complete the hydrogen cycling. Analyzing the plateau pressure provides insights into the difficulty of hydrogen absorption and release—the higher the pressure, the more difficult the absorption or easier the release. Analyzing the slope of the plateau provides information on the continuity of the hydrogen absorption/desorption processes—a smaller slope indicates better continuity. If there is a pressure difference between the hydrogen absorption and release plateaus, it indicates a hysteresis effect in the material’s hydrogen cycling, with a larger difference indicating a more pronounced effect. Additionally, using the Van’t Hoff equation (Equation (1)) to fit the P-C-T curve allows for the calculation of the enthalpy and entropy changes during the hydrogen absorption/desorption reactions.

(1) $$\mathrm{lnP}=\frac{\Delta H}{\mathrm{RT}}-\frac{\Delta S}{R}$$

where Δ*H* represents enthalpy change; Δ*S* denotes entropy change; *P* stands for hydrogen absorption/desorption plateau pressure; *R* is the gas constant; *T* is the testing temperature.

These methods, along with X-ray photoelectron spectroscopy (XPS), Raman spectroscopy, and neutron scattering, are vital for understanding the mechanisms of hydrogen storage, guiding the design and optimization of high-performance materials. Understanding these mechanisms is crucial for the design and optimization of rare-earth-metal-based hydrogen storage materials.

The hydrogen storage performance of rare-earth-metal-based materials is influenced by a multitude of factors. These include the intrinsic properties of the materials such as crystal structure and composition, the specific surface area which affects hydrogen absorption kinetics, and the presence of catalytic additives that can enhance hydrogen uptake and release. Additionally, external conditions such as temperature, pressure, and the cycling stability under repeated hydrogenation and dehydrogenation cycles play crucial roles. Understanding the interplay between these factors is essential for optimizing the efficiency and capacity of hydrogen storage systems based on rare-earth metals:

(1) Composition—it provides information on the crystal structure, lattice parameters, and phase composition of rare-earth-metal-based hydrogen storage materials [47,48]. The specific rare-earth metal and the presence of alloying elements can significantly impact the hydrogen storage capacity, thermodynamics, and kinetics [49];
(2) Microstructure—the particle size, morphology, and surface area of the material can affect the hydrogen absorption and desorption rates, as well as the storage capacity [50]. SEM can also be coupled with energy-dispersive X-ray spectroscopy (EDS) to obtain information on the elemental composition and distribution within the material [49]. The EDS maps provide valuable insights into the homogeneity and distribution of rare-earth elements and other constituents in the hydrogen storage material, which can influence its performance;
(3) Synthesis method—different preparation techniques can result in materials with varying microstructures, purities, and hydrogen storage properties [20];
(4) Operating conditions—the hydrogen pressure, temperature, and cycling conditions can influence the storage capacity, kinetics, and cyclability of the material [21].

Optimization of these factors is essential for the development of high-performance rare-earth-metal-based hydrogen storage materials.

## 3. Hydrogen Storage Performance of Rare-Earth Metals in Alloys

Rare-earth metals, comprising scandium, yttrium, and the lanthanides (elements with atomic numbers 56–70), share similar chemical and physical properties due to their unique electronic structures [50]. These elements and their compounds, particularly oxides and hydrides, exhibit high chemical stability and corrosion resistance, making them suitable for applications in extreme environmental conditions. Moreover, rare-earth elements demonstrate excellent catalytic activity, especially in catalytic cracking and redox reactions, significantly enhancing the efficiency and selectivity of catalysts by providing active sites and regulating reaction processes [51,52,53,54].

The outer electron configuration of rare-earth metals contributes significantly to their metallic bonding characteristics and hydrogen affinity. These valence electrons form the conduction band, which plays a crucial role in determining the nature of metal–hydrogen interactions, including dissociation and absorption processes.

### 3.1. AB_2_ Type

AB_2_-type rare-earth hydrogen storage alloys, with a stoichiometric ratio of 1:2, form complex crystal structures through the combination of rare-earth elements and transition metals. These alloys exhibit relatively stable performance under varying pressure and temperature conditions.

Zhang et al. [55] synthesized AB_2_-type alloys with the composition La_0.8−x_Ce_0.2_Y_x_MgNi_3.4_Co_0.4_Al_0.1_ (x = 0, 0.05, 0.10, 0.15, 0.20) using the melt-spinning technique. They observed that changes in the spinning rate and Y content significantly affected the phase composition. As the spinning rate and Y content increased, the proportion of the LaMgNi_4_ phase increased while the LaNi_5_ phase decreased. The melt-spinning process and the substitution of La with Y also led to significant grain refinement. Furthermore, the addition of Y introduced the MgCu4Sn-type phase.

Research has shown that the occupation of the 4c crystallographic site by Mg significantly impacts the gas–solid hydrogen storage performance of Sm-Mg-Ni-based AB_2_ alloys. In the Sm_0.38_Y_0.10_Mg_0.52_Ni_1.93_Co_0.03_Al_0.05_ alloy, where Mg fully occupies the 4c site, a stable structure with a flat hydrogen absorption/desorption plateau is achieved. This alloy demonstrates a hydrogen absorption capacity of 1.12 wt.% and maintains its MgCu4Sn-type crystal structure after 20 hydrogen absorption cycles, with a capacity retention rate of 94.42%.

While AB_2_-type alloys exhibit good cyclic stability, they face challenges such as relatively slow hydrogen absorption/desorption kinetics and susceptibility to hydrogen embrittlement after prolonged cycling. These limitations restrict their applications in certain industrial sectors. To address these issues, further optimization through microstructural adjustment or elemental doping is necessary. Such improvements could potentially enhance the kinetics and mitigate embrittlement, expanding the range of applications for these alloys in hydrogen storage technologies.

### 3.2. AB_3_ Type

AB3-type rare-earth hydrogen storage alloys represent an emerging class of materials characterized by their excellent hydrogen absorption capacity under low-pressure conditions. The unique crystal structure and chemical composition of these alloys offer greater flexibility for performance tuning, while their stable thermodynamic properties make them suitable for various hydrogen storage applications.

Jiang et al. [56] synthesized Re_2_Mg(Ni_0.7−x_Co_0.2_Mn_0.1_Al_x)9_ (x = 0–0.04) alloys using induction melting. X-ray diffraction analysis revealed that these alloys primarily consist of LaNi_5_ and (La,Mg)_2_Ni_7_ phases, with a minor LaNi2 phase. As the Al content increased, the maximum hydrogen storage capacity at 303 K decreased from 1.16 wt.% (x = 0) to 0.99 wt.% (x = 0.04). Concurrently, the thermodynamic stability and structural disorder of the hydrides increased, as evidenced by changes in the plateau pressure and slope. In a related study, Xin et al. [57] prepared La_(0.65−x)_Y_x_Mg_1.32_Ca_1.03_Ni_9_ (x = 0, 0.05, 0.20, 0.40, 0.60) alloys via induction melting. They discovered that modulating the Y content allowed for precise control of the hydrogen absorption plateau pressure. The effective hydrogen absorption capacity was significantly enhanced when the Y content reached an optimal level. Notably, La_0.65_Mg_1.32_Ca_1.03_Ni_9_ exhibited a hydrogen storage capacity of 1.831 wt.% under 10 MPa H_2_, releasing 1.701 wt.% of absorbed hydrogen at room temperature. At room temperature, La_0.60_Y_0.05_Mg_1.32_Ca_1.03_Ni_9_ and La_0.45_Y_0.20_Mg_1.32_Ca_1.03_Ni_9_ demonstrated hydrogen absorption capacities of 1.784 wt.% and 1.753 wt.%, respectively. However, further increases in Y content led to a reduction in the AB3 phase, resulting in a significant decline in hydrogen storage capacity. For instance, La_0.05_Y_0.60_Mg_1.32_Ca_1.03_Ni_9_ absorbed only 1.292 wt.% hydrogen under 10 MPa H_2_ at room temperature.

While research on AB_3_-type alloys is still in its early stages, the unique crystal structure of these materials offers substantial potential for flexible tuning of performance characteristics. Further experimental studies focusing on composition optimization, microstructure control, and the relationship between structure and hydrogen storage properties will be essential for broadening the application of these promising materials in hydrogen storage technologies.

### 3.3. AB_5_ Type

AB5-type rare-earth hydrogen storage alloys, with LaNi_5_ as the prototypical example, are among the most extensively researched and applied hydrogen storage materials. In these alloys, the A-site is typically occupied by rare-earth metals (e.g., lanthanum and cerium), while the B-site is filled by transition metals (e.g., nickel and cobalt). These alloys have garnered significant attention due to their excellent reversible hydrogen storage performance, high hydrogen absorption capacity, and moderate hydrogen absorption/desorption pressure conditions.

**Figure 3:**
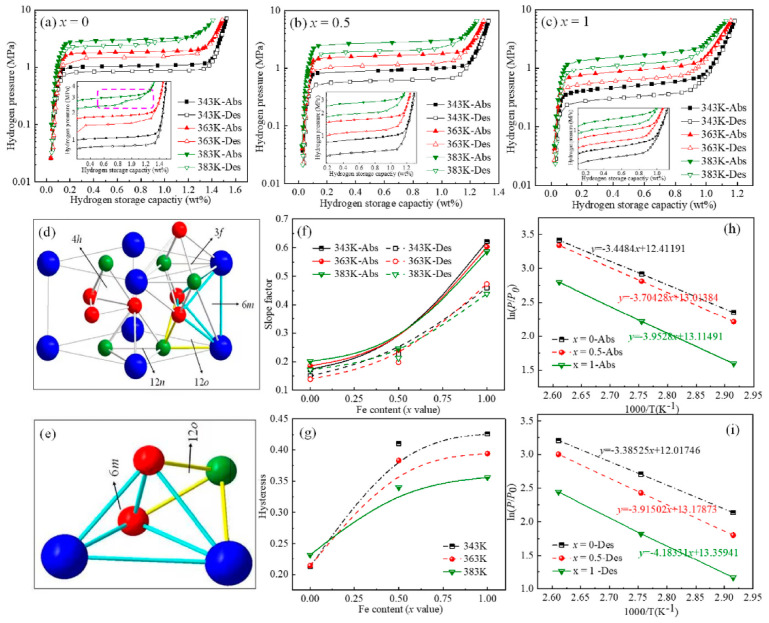
LaNi_5−x_Fe_x_ (x = 0, 0.5, 1) alloys’ (**a**) P–C-T curves at 343 K, (**b**) 363 K, and (**c**) 383 K; (**d**) five lattice interstitial positions and (**e**) enlarged view of 6m and 12o interstitial sites of LaNi_5_ alloy (Blue is La-1a, Green is Ni1-2c, Red is Ni2-3g); (**f**) the slope values; (**g**) hysteresis values of LaNi_5−x_Fe_x_ (x = 0, 0.5, 1) alloys at 343 K, 363 K, and 383 K; (**h**) Van’t Hoff plots of hydrogen absorption and (**i**) desorption [58].

Despite their promise, the overall hydrogen storage performance of AB5-type alloys, particularly their long-term durability, requires further optimization. Zhu et al. [55] investigated the microstructure and long-term hydrogen absorption/desorption performance of La_5−x_Ce_x_Ni_4_Co (x = 0.4, 0.5) and La_5−y_Y_y_Ni_4_Co (y = 0.1, 0.2) alloys. The addition of Ce or Y increased the c/a ratio of the unit cell, significantly improving the alloy’s resistance to pulverization and structural stability. Ce and Y incorporation also eliminated the secondary plateau in the alloy’s pressure–composition–temperature (P-C-T) curve, enhancing its application efficiency. Notably, the La_4.5_Ce_0.5_Ni_4_Co alloy exhibited an extended P-C-T plateau, reaching 95.5% of full capacity, while the other three alloys ranged from 67% to 77%. Cheng et al. [58] studied the effect of Fe on the long-term hydrogen storage performance of LaNi_5−x_Fex (x = 0, 0.5, 1) alloys. As Fe content increased (Figure 3a–c), the proportion of the non-hydrogen-absorbing (Fe,Ni) phase also increased, resulting in decreased hydrogen storage capacity. Fe substitution for Ni effectively suppressed plateau splitting observed in LaNi_5_. As shown in Figure 3d,e, the researchers proposed a mechanism for hydrogen occupation in interstitial sites: 4h sites (α-phase), 3f, 12n, and 12o sites (β-phase, 3 ≤ y ≤ 4), and 6m sites (γ-phase, y > 6). Fe substitution, with its larger atomic radius, increased unit cell volumes, alleviating compression effects and reducing plateau splitting (Figure 3f,g). Additionally, Fe substitution induced lattice strain, increasing atomic disorder and hysteresis while enhancing metal hydride stability through increased enthalpy change in the hydrogen absorption/desorption reaction (Figure 3h,i). Wang et al. [59] prepared AB_5_-type La_0.6_Mg_x_Ni_3.45_Nd_0.1_ (x = 0.2, 0.25, 0.3, 0.35, 0.4) alloys using suction casting (SC) and conventional as-cast (AC) methods. SC alloys exhibited more uniform morphology and structure, with higher hydrogen storage capacity (~1.63 wt.%) compared to AC alloys (~1.57 wt.%). The La_0.6_Mg_0.3_Ni_3.45_Nd_0.1_ SC alloy demonstrated a hydrogen capacity retention rate of 90.86% after 300 cycles at 298 K, surpassing the AC alloy’s performance.

AB_5_-type alloys, particularly LaNi_5_, are recognized for their mature technology and broad application prospects, excelling in cycle life, hydrogen absorption/desorption kinetics, and operational stability. However, their performance limitations at elevated temperatures restrict their application range, especially for ambient- or lower-temperature hydrogen storage. Further material modifications are necessary to enhance their hydrogen storage capabilities and broaden their operational temperature range.

### 3.4. Other Rare-Earth Hydrogen Storage Alloys

Beyond the conventional AB_2_, AB_3_, and AB_5_-type alloys, rare-earth hydrogen storage materials with alternative stoichiometries have garnered increasing attention. These alloys, including A_2_B_7_ and A_5_B_19_ types, typically feature more complex compositions and crystal structures. By fine-tuning the ratio of A-site to B-site metal elements and incorporating additional transition metals or rare-earth elements, researchers can achieve more versatile hydride phase formation and performance optimization.

**Figure 4:**
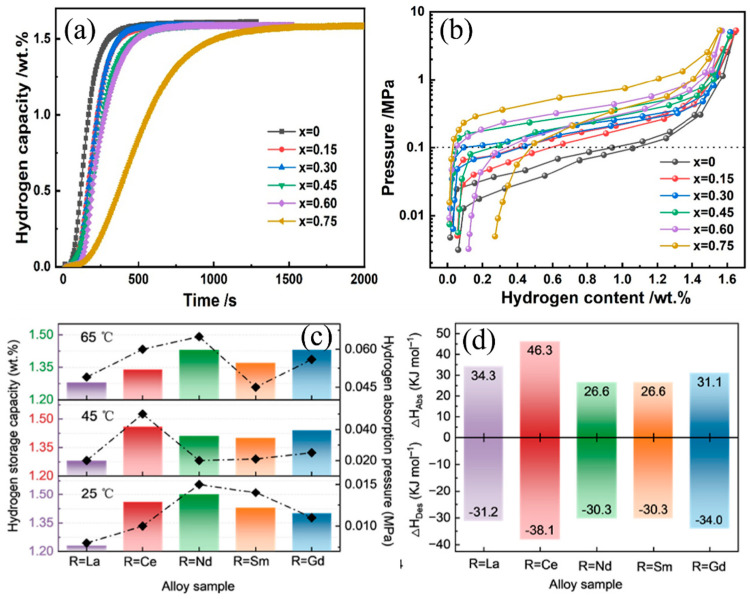
La_1−x_Ce_x_Y_2_Ni_10.95_Mn_0.45_ alloy’s (**a**) hydrogen absorption kinetic curves, (**b**) PCT curves at 298 K [60]; La_0.67_R_0.05_Y_0.13_Mg_0.15_Ni_3.70_Al_0.15_ (R = La, Ce, Nd, Sm, Gd) alloys’ (**c**) hydrogen storage capacity and hydrogen absorption platform pressure at different temperatures and (**d**) enthalpy values during hydrogenation and dehydrogenation [61].

A_2_B_7_-type alloys have shown particular promise in hydrogen storage applications. Liu et al. [60] investigated the phase structure and gas–solid hydrogen storage performance of La_1−x_Ce_x_Y_2_Ni_10.95_Mn_0.45_ (x = 0, 0.15, 0.30, 0.45, 0.60, 0.75) alloys. Their study revealed that during initial hydrogen absorption, the absorption rate gradually decreased with increasing Ce content (Figure 4a). The La_0.55_Ce_0.45_Y_2_Ni_10.95_Mn_0.45_ composition exhibited the highest hydrogen absorption capacity of 1.61 wt.%, maintaining a 97.89% capacity retention rate after 100 cycles, with a hydrogen absorption plateau pressure of 0.23 MPa. This superior performance was attributed to the Ce-induced phase transformation from Gd_2_Co_7_ to LaNi_5_, which simplified the alloy’s hydrogen absorption/desorption plateau from dual to single. However, higher Ce content also increased residual hydrogen during desorption, indicating a trade-off in performance optimization (Figure 4b). A_5_B_19_-type alloys have emerged as potential alternatives to A_2_B_7_-type materials. Zhang et al. [61] examined the effects of A-site elements (Ce, Nd, Sm, and Gd) on the crystal structure and hydrogen storage performance of A_5_B_19_-type La_0.72_Y_0.13_Mg_0.15_Ni_3.70_Al_0.15_ alloys. As shown in Figure 4c that while different elemental substitutions affected hydrogen storage capacity at various temperatures, Ce incorporation rendered the capacity more temperature-sensitive, with variations up to 0.08 wt.%. Nd addition yielded the highest hydrogen storage capacity of 1.5 wt.%. Notably, alloys containing Nd and Sm showed more stable capacities across temperatures, correlating with their lower enthalpy changes during hydrogen absorption and desorption reactions. The same research team also investigated the impact of substituting Ni with Mn and Fe [62]. Comparing La_0.72_Y_0.13_Mg_0.15_Ni_3.65_Al_0.15_Fe_0.05_ to its Mn-containing counterpart, they observed an increase in gas–solid hydrogen storage capacity of 1.44 wt.% and a change in hydrogen absorption enthalpy of −22.9 kJ mol^−1^ (Fiugre 4d). These findings underscore the significant influence of both A-site and B-site substitutions on hydrogen storage properties.

These non-standard stoichiometric alloys often exhibit unique hydrogen storage characteristics, including higher capacities, improved kinetics, or broader operational temperature and pressure ranges. Their hydride phases tend to offer enhanced stability and reversibility, making them suitable for specific applications. However, the complexity of their compositions and the diversity of interaction mechanisms present significant challenges in development and optimization. Further in-depth research is necessary to fully realize their practical potential in hydrogen storage applications.

## 4. Optimization Strategies for Rare-Earth-Metal-Based Hydrogen Storage Materials

To enhance the hydrogen storage performance of alloys, various optimization strategies have been employed. These strategies aim to improve the storage capacity, kinetics, and thermodynamics of the materials, as well as their stability and cyclability.

### 4.1. Elemental Substitution

Doping rare-earth metals with other elements is an effective strategy to enhance their hydrogen storage properties. The dopants can modify the electronic structure, crystal lattice, and surface properties of the host material, leading to improved hydrogen absorption/desorption kinetics and thermodynamics [63]. For instance, doping LaNi_5_ with rare-earth elements such as Ce, Pr, and Nd has been reported to lower the hydrogen desorption temperature and increase the reversible hydrogen storage capacity [64]. Surface modification of rare-earth-metal-based hydrogen storage materials with catalytic nanoparticles or coatings is another promising approach to enhance their performance. The surface modifiers can facilitate the dissociation of hydrogen molecules, improve the hydrogen diffusion kinetics, and protect the material from oxidation and poisoning [65]. Various surface modification techniques, such as catalytic nanoparticle deposition, polymer coating, and organic ligand functionalization, have been explored for rare-earth-metal-based hydrogen storage materials [66].

**Figure 5:**
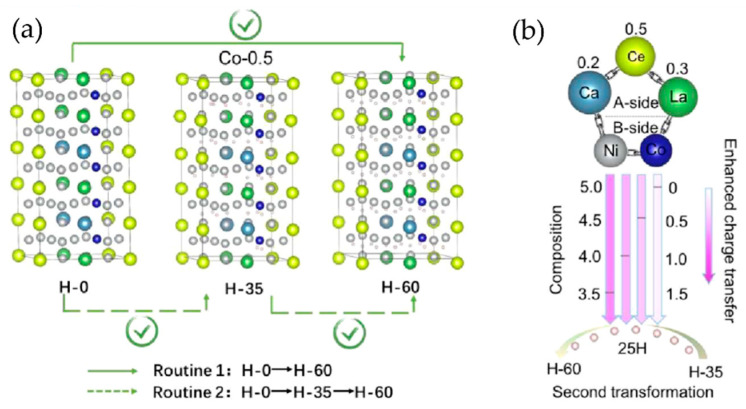
La_0.3_Ce_0.5_Ca_0.2_Ni_5−x_Co_x_ (x = 0−1.5) based alloy’s (**a**) phase transformation process for Co-0.5 alloy and (**b**) charge synergy and transfer during the second transformation [70].

For example, substituting rare-earth metal hydrides with transition metals, such as Ni, Co, or Fe, has been shown to enhance the hydrogen absorption and desorption kinetics, as well as the reversibility of the storage process [67]. The transition metal can act as catalysts, facilitating the dissociation of hydrogen molecules and the formation of hydride phases [68]. They can also modify the thermodynamic stability of the hydrides, allowing for lower desorption temperatures and pressures [69]. Zhou et al. [70] synthesized single-phase AB_5_-type La-Ce-Ca-Ni-Co alloys with homogeneous elemental distribution using induction levitation melting. They investigated the composition La_0.3_Ce_0.5_Ca_0.2_Ni_5−x_Co_x_ (x = 0, 0.5, 1.0, 1.5), observing that increasing Co content induced a stepwise phase transition. This transition significantly improved pressure hysteresis characteristics, although the desorption equilibrium pressure did not exhibit a monotonic decrease. First-principles calculations revealed that the enhancement of charge synergistic effects and increased charge transfer facilitated the stepwise phase transition from a dynamically stable pathway to a thermodynamically stable one. As shown in Figure 5a, the researchers proposed that the term “transitional phase transition” more accurately describes a “dynamic stepwise phase transition,” where the phase change dynamically realizes a thermodynamically stepwise transition (Path 1) via a dynamic pathway (Path 2). At a Co content of 1.5, the energy change (ΔE) for the second transition became negative (−0.1452 eV), indicating the overcoming of the initial reaction energy barrier. Consequently, the phase transition preferentially occurred through Path 2, validating the dynamic stepwise phase transition model in the Co-1.5 alloy. The reaction energy barrier of this second transition emerged as the critical factor governing the phase transition’s existence and behavior. Atomic charge analysis demonstrated that increasing Co content led to more pronounced atomic charge transfer, correlating well with the observed ΔE trends. This enhanced charge transfer promotes hydrogen atom fixation and stabilizes the second phase transition, thereby improving the alloy’s overall hydrogen storage performance (Figure 5b). The La_0.25_Ce_0.55_Ca_0.2_Ni_4.5_Co_0.5_ composition exhibited optimal performance, with a saturated hydrogen storage capacity of 1.52 wt.%, a desorption equilibrium pressure of 10.68 MPa at 90 °C, and excellent cyclic stability, demonstrating its potential for durable hydrogen storage applications. This study highlights the complex interplay between elemental composition, electronic structure, and phase transition behavior in rare-earth-based hydrogen storage alloys, providing valuable insights for the design of high-performance materials.

Substitution of the rare-earth metal with other elements, such as Mg or Ca, can also lead to improved hydrogen storage properties. These substitutions can alter the crystal structure and the size of the interstitial sites, influencing the hydrogen storage capacity and the thermodynamic properties of the material [66]. Ternary alloys, composed of three different metal elements, can significantly enhance hydrogen storage capacity and cyclic stability through optimizing the element ratios. Their main advantages in hydrogen storage include optimized performance, ease of processing, and high thermodynamic stability. Additionally, ternary alloys composed of rare-earth elements (such as Y, Zn, and Gd), magnesium, and transition metals (such as Al, Zn, and Ni) easily form long-period stacking ordered (LPSO) structures. This is a special intermetallic compound structure characterized by long-period ordered arrangements, typically with repeat units of 10 to 30 atomic layers. The highly ordered stacking sequences and unique microstructures of LPSO significantly enhance the material’s hydrogen storage capacity, hydrogen absorption and desorption kinetics, and cycling stability. The LPSO phase provides more insertion sites for hydrogen atoms, increasing the hydrogen storage capacity, and improves absorption and desorption kinetics through rapid hydrogen diffusion paths. Furthermore, the stability of the LPSO structure reduces structural degradation during hydrogenation and dehydrogenation cycles, extending the cycle life while lowering the hydrogenation and dehydrogenation temperatures, thus improving hydrogen storage efficiency. Zhang et al. [71] fabricated Mg_96_Y_2_Zn_2_ alloys with a long LPSO structure, featuring fine α-Mg dendrites and uniformly distributed Zn and Y regions between the dendrites. After hydrogen absorption/desorption, the alloy decomposed into YH_2_ + Mg(Zn) composites. During the reaction, YH_2_ transformed into YH_3_, creating a “hydrogen pump” effect. Combined with the catalytic action of MgZn_2_, this composite could absorb 5.36 to 5.79 wt.% H_2_ at 300 °C and 2 MPa and desorb hydrogen at lower temperatures. Pan et al. [72] prepared Mg-9.1Y-1.8Zn alloys in different states using semi-continuous casting and extrusion processes. The three different states of Mg-9.1Y-1.8Zn alloys were all composed of Mg phases and LPSO phases. After hydrogen absorption, the LPSO phase fractured and decomposed, in situ generating catalytically active YHc (c = 2.3) nanohydrides. Additionally, after forging, the core of the alloy exhibited numerous nanocrystals with average sizes between 80 and 200 nm, increasing the specific surface area of the alloy and demonstrating excellent hydrogen absorption kinetics.

Ni is also commonly used for optimizing Mg-Y alloys. Zhang et al. [73] prepared nanocrystalline and amorphous Mg-5Ni-3La (at.%) alloys using rapid solidification. The enthalpy values of the Mg-H_2_ system for the nanocrystalline and amorphous alloys were −74.0 ± 1.2 kJ/mol and −66.1 ± 3.4 kJ/mol, respectively, which are significantly lower than the enthalpy value of −77.7 ± 0.3 kJ/mol for the as-cast alloy. Zhang et al. [74] fabricated nanocrystalline and amorphous alloys with compositions of Mg_20−x_Y_x_Ni_10_ (x = 0, 1, 2, 3, and 4) using mechanical milling. The results showed that with increasing Y content, the alloy phase structure changed significantly. When x = 1, Y replaced Mg to form the secondary phase YMgNi_4_, while the primary phase Mg_2_Ni remained unchanged. However, with further increase in Y content, the primary phase transformed to YMgNi_4_, which is the main reason for the changes in hydrogen storage capacity of the alloys. Yu [75] prepared Mg_95−X_-Ni_x_-Y_5_ (x = 5, 10, 15) alloys using vacuum induction melting. Tests revealed that the YH_3_ phase formed in situ after hydrogenation was uniformly dispersed in the parent alloy but did not decompose during dehydrogenation, only providing a catalytic effect on the reversible reaction of Mg and Mg_2_Ni phases. With the increase in Ni content, the enthalpy of the magnesium-based hydrogen storage alloy reactants was effectively reduced to 84.5, 69.1, and 63.5 kJ/mol H_2_. Additionally, the hydrogen absorption and desorption kinetics were significantly improved. Notably, the Mg_80_Ni_15_Y_5_ alloy could release approximately 5.0 wt.% H_2_ within 5 min at 320 °C, nearly matching the dehydrogenation capacity of Ni_5_ alloy at 360 °C, and the dehydrogenation activation energy was reduced to 67.0 kJ/mol. After complete dehydrogenation, the alloy could absorb 5.4 wt.% H_2_ within 1 min at 200 °C and 3 MPa.

**Figure 6:**
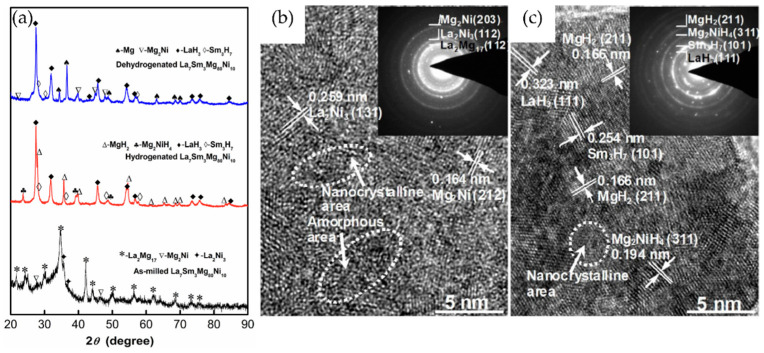
The milled La_7_Sm_3_Mg_80_Ni_10_ alloy’s (**a**) XRD profiles before and after being hydrogenated and dehydrogenated; HRTEM (**b**) as-milled and (**c**) hydrogenated [77].

Multi-element alloys are composed of multiple metal elements, allowing for a wide range of performance adjustments due to their complex composition design. These alloys exhibit high-temperature stability, multifunctionality, and excellent overall properties. Yao et al. [76] prepared Mg-Ni-Gd-Y-Zn-Cu alloys by using direct chill casting. The as-cast alloy consisted of a primary α-Mg phase and Mg_2_Ni/LPSO-layered eutectics. Compared to the as-cast state, the extruded alloy showed higher hydrogen absorption capacity and faster hydrogen absorption/desorption kinetics. At 360 °C under 20 bar H_2_ pressure, the extruded alloy could absorb 4.50 wt.% H_2_ within 5 min. Furthermore, at 360 °C under 1 bar H_2_ pressure, it could desorb 4.59 wt.% H_2_ within 2 min, achieving 94% of the initial hydrogen absorption rate. Qi et al. [77]. prepared La_7_Sm_3_Mg_80_Ni_10_ quaternary alloys using casting and ball milling methods. XRD analysis showed that ball milling broadened the diffraction peaks, reducing both particle and grain sizes, thus exhibiting typical nanocrystalline and amorphous characteristics while also altering their surface properties (Figure 6a). Combined with HRTEM (Figure 6b,c) analysis, it was revealed that before hydrogen absorption, the alloy consisted of three phases: a primary La_2_Mg_17_ phase and secondary Mg_2_Ni and LaNi_3_ phases. The initial hydrogen desorption temperature was 547.4 K, and the ball-milled alloy absorbed 4 wt.% H_2_ within just 50 **s** at 553 K. Zhang et al. [78] improved the hydrogen storage performance of La−Y−Mg−Ni alloys by preparing amorphous and nanocrystalline structures of La_1.7_Y_0.3_Mg_16_Ni alloys using ball milling for 5 to 30 h. With the extension of milling time, the crystallinity, grain size, and particle size of the alloy decreased, while the amorphous phase increased. Under the dual regulation of nanocrystalline and amorphous phases, the hydrogen storage kinetics initially improved and then declined. The alloy milled for 15 h exhibited the best hydrogen absorption/desorption kinetics, absorbing 3.10 wt.% hydrogen within 10 min at 373 K, with the lowest dehydrogenation activation energy of 71.2 kJ/mol.

### 4.2. Composite Materials

The development of composite materials is a versatile approach for optimizing the hydrogen storage properties of rare-earth-metal-based systems. By combining the rare-earth-metal-based material with other components, such as metal hydrides, carbon nanostructures, or metal–organic frameworks, synergistic effects can be achieved, leading to enhanced storage capacity, kinetics, and thermodynamics [79].

One example of a composite material is the integration of rare-earth-metal-based materials with carbon nanostructures, such as graphene, carbon nanotubes, or porous carbon. The carbon nanostructures can provide a high surface area and a conductive network for hydrogen diffusion, enhancing the kinetics of hydrogen absorption and desorption [80]. They can also act as a structural support, preventing the aggregation and sintering of the rare-earth-metal-based particles during cycling [81]. Ball milling Eu_2_O_3_@C with Mg_96_La_3_Ni alloy significantly improved the dehydrogenation kinetics of Mg_96_La_3_Ni, with the best effect observed at a Eu_2_O_3_@C content of 2 wt.% [82]. At 360 °C, 77.3% of the maximum hydrogen capacity could be absorbed within 2 min, and 3 wt.% H_2_ could be released in just 2 min. Additionally, Eu_2_O_3_@C significantly reduced the dehydrogenation activation energy of the alloy to 106.3 kJ/mol and lowered the endothermic peak temperature to 347 °C. Ball milling Mg_96_La_3_Ni alloy with varying amounts of CeO_2_@C resulted in significant refinement of the magnesium-based alloy, increasing its specific surface area and the number of active sites [83]. When the addition amount was 5 wt.% CeO_2_@C, at 360 °C, the alloy could absorb 78.1% of its maximum hydrogen capacity within 2 min and release an equivalent amount of hydrogen within 5 min, with the dehydrogenation activation energy reduced to 107.33 kJ/mol H_2_. This improvement was primarily due to the synergistic catalytic effect of CeO_2_ nanoparticles, which mainly promoted nucleation, and the carbon matrix, which facilitated hydrogen diffusion. The Mg_96_La_3_Ni alloy with different proportions of Yb_2_O_3_@C also resulted in significant refinement of the magnesium-based alloy, increasing its specific surface area and the number of active sites [84]. When the Yb_2_O_3_@C content was 3 wt.%, it significantly reduced the dehydrogenation activation energy of the alloy. Milling Mg_96_La_3_Ni alloy with 1 wt.% GdO@C revealed that the composite material could quickly absorb a large amount of hydrogen (84.4 wt.% of its maximum capacity) within 2 min at 360 °C and release a small amount of hydrogen (3 wt.%) in 2 min [85]. Doping the alloy with GdO@C reduced the dehydrogenation activation energy barrier (to 106.3 kJ/mol), with the hydrogen desorption peak temperature appearing at 334 °C. Notably, the thermodynamic properties of the composite material did not improve, with the enthalpy change remaining constant at 77.1 kJ/mol.

Another promising class of composite materials is the combination of a rare-earth metal hydride with a light metal hydride, such as MgH_2_ or LiBH_4_. The light metal hydride can contribute to the overall hydrogen storage capacity, while the rare-earth metal hydride can act as a catalyst, improving the hydrogen absorption and desorption kinetics of the composite [86]. The interaction between the two components can also modify the thermodynamic stability of the hydrides, allowing for lower desorption temperatures and pressures [87,88]. Metal–organic frameworks (MOFs) have also been explored as components in rare-earth-metal-based composite materials [89]. MOFs are highly porous structures composed of metal ions or clusters connected by organic linkers, offering a large surface area and tunable pore sizes [90]. By incorporating rare-earth-metal-based nanoparticles or clusters within the MOF structure, the hydrogen storage capacity and kinetics can be enhanced [91]. The confinement effect of the MOF can also modulate the thermodynamics of the hydrogen storage process, leading to improved reversibility and cyclability [92].

The rational design and synthesis of composite materials require a deep understanding of the structure–property relationships and the interfacial interactions between the components. Advanced characterization techniques, such as in situ X-ray absorption spectroscopy, neutron scattering, and electron microscopy, are crucial for unraveling the mechanisms underlying the enhanced hydrogen storage performance of these materials [93].

### 4.3. Nanostructure Design

Nanostructuring is a powerful approach for enhancing the hydrogen storage performance of rare-earth-metal-based materials. By reducing the particle size to the nanoscale, the surface area and the number of active sites for hydrogen absorption and dissociation can be significantly increased [94]. Nanostructuring can also shorten the diffusion path length for hydrogen atoms, leading to improved absorption and desorption kinetics [93].

**Figure 7:**
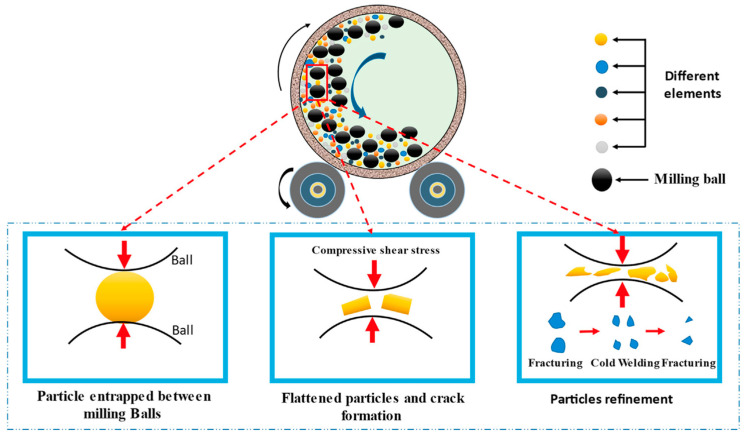
Schematic of the mechanical ball milling process [97,98].

Various nanostructures, such as nanoparticles, nanorods, nanosheets, and nanoporous materials, have been explored for alloys’ hydrogen storage [95]. These nanostructures can be prepared using techniques such as ball milling, solvothermal synthesis, electrochemical deposition, and templating methods [96]. As shown in Figure 7, during the milling process, the balls repeatedly collide with the powder particles, causing a continuous cycle of welding, fracturing, and re-welding [97,98]. This process leads to a reduction in particle size, the creation of new surfaces, and the introduction of defects and strain [97,98].

Nanoconfinement is another effective strategy for improving the hydrogen storage properties of rare-earth-metal-based materials. By confining the material within a nanoporous scaffold, such as carbon nanotubes, mesoporous silica, or metal–organic frameworks, the thermodynamic and kinetic properties can be tuned [99]. The nanoconfinement can stabilize the hydride phases, reduce the desorption temperature, and prevent particle aggregation during cycling [100].

### 4.4. Surface Modification and Coating

Surface modification and coating are effective strategies for enhancing the hydrogen storage performance of an alloy. By tailoring the surface chemistry and morphology, the hydrogen absorption and desorption kinetics, as well as the stability and cyclability of the materials, can be improved [101].

One approach is the deposition of rare-earth-metal-based material onto the surface of the alloys [102]. These catalytic nanoparticles can facilitate the dissociation of hydrogen molecules and the spillover of hydrogen atoms onto the material surface, enhancing the hydrogen absorption kinetics [103]. The catalytic effect can also lower the activation energy for hydrogen desorption, allowing for faster release of hydrogen under milder conditions [66].

**Figure 8:**
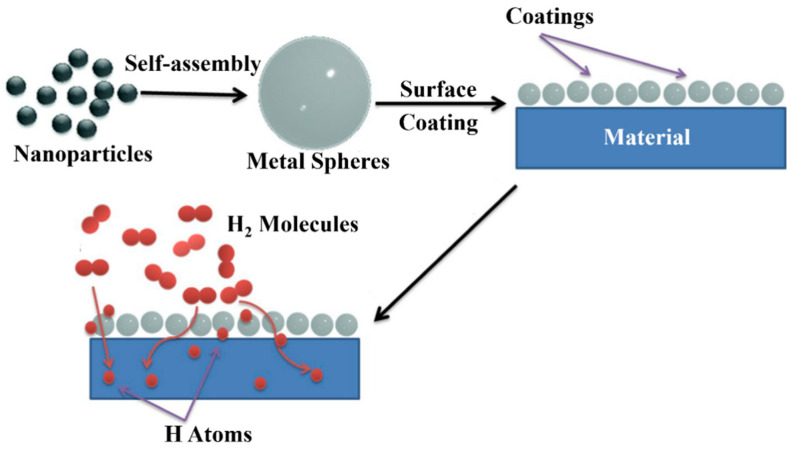
Schematic of coating process and mechanism [103].

Another approach is the application of protective coatings, such as polymer or metal oxide layers, onto the surface of the rare-earth-metal-based material. As shown in Figure 8, the coatings can act as a barrier against oxidation and moisture, preventing the degradation of the hydrogen storage properties over time [104]. They can also modulate the surface chemistry and the hydrogen absorption/desorption behavior, leading to improved kinetics and cyclability [104].

Surface functionalization with organic ligands or functional groups is another promising strategy for enhancing the hydrogen storage performance of rare-earth-metal-based materials. By grafting suitable ligands onto the surface, the electronic structure and the hydrogen binding energy can be tuned, leading to improved thermodynamics and kinetics [82]. The ligands can also act as spacers, preventing particle agglomeration and maintaining a high surface area during cycling [83].

## 5. Conclusions and Perspectives

Rare-earth-metal-based hydrogen storage materials have emerged as a promising class of materials for enabling the widespread adoption of hydrogen as a clean and sustainable energy carrier. With their unique properties, such as high hydrogen affinity, reversible hydrogen absorption/desorption, and tunable thermodynamics, these materials offer significant potential for the development of efficient and compact hydrogen storage systems.

This comprehensive review has provided an in-depth analysis of the recent advancements, challenges, and future prospects of rare-earth-metal-based hydrogen storage materials. The fundamental aspects, including the electronic structure, hydrogen storage mechanisms, and thermodynamic and kinetic properties, have been discussed in detail. The various synthesis methods, such as ball milling, solvothermal synthesis, and chemical vapor deposition, have been reviewed, highlighting their advantages and limitations in producing high-performance materials.

Despite the significant progress made in the development of rare-earth-metal-based hydrogen storage materials, several challenges remain to be addressed for their practical application. At the same time, these challenges present opportunities for further research and innovation in this field.

### 5.1. Current Limitations and Issues

One of the main challenges facing rare-earth-metal-based hydrogen storage materials is their relatively low actual hydrogen storage capacity compared to the targets set by the U.S. Department of Energy (DOE) for automotive applications. While some rare-earth metal hydrides, such as LaNi_5_H_6_, can achieve high volumetric hydrogen densities, their actual capacities are limited by the heavy atomic mass of the rare-earth metals. Strategies such as nanostructuring, alloying, and compositing have been employed to improve the actual capacity, but further advancements are needed to meet the DOE targets.

Another issue is the slow kinetics of hydrogen absorption and desorption in some rare-earth-metal-based materials, particularly at low temperatures. The high activation energy barriers for hydrogen dissociation and diffusion can limit the practical application of these materials, especially in on-board hydrogen storage systems. Surface modification, catalytic doping, and nanostructuring have shown promise in enhancing the kinetics, but further optimization is required to achieve fast and reversible hydrogen storage under mild conditions.

The thermodynamic stability of some rare-earth metal hydrides is also a challenge, as it can lead to high desorption temperatures and pressures, making them unsuitable for practical applications. Strategies such as alloying, nanoconfinement, and destabilization with reactive additives have been explored to tune the thermodynamics, but finding the right balance between stability and reversibility remains a challenge.

The long-term cycling stability and reversibility of rare-earth-metal-based hydrogen storage materials are also critical issues that need to be addressed. The repeated absorption and desorption of hydrogen can lead to structural changes, particle aggregation, and loss of storage capacity over time. Strategies such as nanoconfinement, surface coating, and compositing with stable matrices have been employed to improve the cycling performance, but further enhancements are necessary for practical applications.

### 5.2. Strategies for Performance Enhancement

To overcome the limitations and enhance the performance of rare-earth-metal-based hydrogen storage materials, several strategies have been proposed and explored:

Rational design of alloys and composites: by carefully selecting the composition and stoichiometry of rare-earth metal alloys and composites, the hydrogen storage properties can be optimized. Computational methods, such as density functional theory (DFT) calculations and machine learning, can aid in the prediction and screening of promising alloy and composite compositions, guiding experimental efforts.

Advanced nanostructuring techniques: the development of novel nanostructuring techniques, such as 3D printing, templating, and self-assembly, can enable the fabrication of rare-earth-metal-based materials with precise control over the size, shape, and porosity. These advanced nanostructures can offer enhanced surface area, reduced diffusion lengths, and improved kinetics for hydrogen storage.

Surface engineering and catalysis: the rational design of surface coatings, catalytic nanoparticles, and functional ligands can significantly enhance the hydrogen absorption/desorption kinetics and the cycling stability of rare-earth-metal-based materials. The surface engineering approach can also help to protect the materials from oxidation and contamination, improving their long-term performance.

In situ characterization and mechanistic studies: advanced in situ characterization techniques, such as X-ray absorption spectroscopy, neutron scattering, and environmental TEM, can provide valuable insights into the dynamic processes occurring during hydrogen storage. These studies can help to elucidate the mechanisms underlying the enhanced performance of optimized materials and guide further improvements.

Machine learning and high-throughput screening: the application of machine learning algorithms and high-throughput computational screening can accelerate the discovery and optimization of rare-earth-metal-based hydrogen storage materials. By leveraging large datasets and advanced computational tools, promising compositions and structures can be identified and prioritized for experimental validation, reducing the time and cost associated with materials development.

### 5.3. Techno-Economic Barriers and Opportunities

For the widespread adoption of rare-earth-metal-based hydrogen storage materials in practical applications, such as fuel cell vehicles and stationary power systems, several techno-economic barriers need to be overcome:

Cost: the high cost of rare-earth metals and the complex processing techniques required for the synthesis of optimized hydrogen storage materials can hinder their large-scale production and deployment. Strategies such as recycling, process optimization, and the use of abundant precursors need to be explored to reduce the overall cost of these materials.

Scalability: the production of rare-earth-metal-based hydrogen storage materials at industrial scales requires the development of scalable and efficient synthesis methods. The challenges associated with maintaining the desired nanostructure, composition, and performance during scale-up need to be addressed through process engineering and quality control measures.

Safety and regulations: the safe handling, storage, and transportation of rare-earth-metal-based hydrogen storage materials are critical for their practical application. The development of appropriate safety protocols, regulations, and infrastructure is necessary to ensure the safe and reliable use of these materials in hydrogen storage systems.

Life cycle assessment and sustainability: the environmental impact and sustainability of rare-earth-metal-based hydrogen storage materials need to be carefully evaluated throughout their life cycle, from raw material extraction to end-of-life disposal. The development of eco-friendly and sustainable production methods, as well as effective recycling and reuse strategies, is crucial for the long-term viability of these materials.

Despite these challenges, the increasing global demand for clean and sustainable energy solutions presents significant opportunities for the development and commercialization of rare-earth-metal-based hydrogen storage materials. The ongoing research and innovation in this field, coupled with supportive government policies and industry collaborations, can help to overcome the techno-economic barriers and accelerate the adoption of these materials in practical applications.

### 5.4. Future Research Directions and Prospects

Rare-earth-metal-based hydrogen storage materials offer promising research and development opportunities. Key directions include developing multifunctional materials that combine hydrogen storage with catalysis, sensing, or thermal management, thus enhancing performance and versatility. Hybrid storage systems, which integrate rare-earth-metal-based materials with other technologies like compressed gas or liquid hydrogen, can improve capacity, kinetics, and versatility, overcoming individual limitations.

Advancements in real-time studies and advanced characterization techniques, such as in situ neutron scattering, X-ray absorption spectroscopy, and TEM, provide valuable insights into the dynamic processes of hydrogen storage. These techniques help with understanding structure–property relationships and guiding the design of optimized materials. Additionally, integrating advanced computational methods like DFT calculations, molecular dynamics simulations, and machine learning accelerates the discovery and optimization of rare-earth-metal-based hydrogen storage materials by predicting novel compositions and structures and elucidating the mechanisms of hydrogen storage.

Sustainability is crucial for the long-term viability of these materials. Exploring bio-derived precursors, green synthesis methods, and closed-loop life cycle strategies enhances environmental sustainability. Recent advancements in materials design, nanostructuring, surface engineering, and real-time characterization have deepened our understanding of the structure–property relationships and mechanisms underlying enhanced hydrogen storage performance. Future research should focus on designing multifunctional materials, developing hybrid storage systems, and exploring sustainable production methods. Collaborative efforts among researchers, industry partners, and policymakers are essential to overcoming barriers and realizing the potential of these materials in practical hydrogen storage applications, thereby contributing significantly to the hydrogen-based economy.

As the field of rare-earth-metal-based hydrogen storage continues to evolve, it is expected that new discoveries, innovative strategies, and breakthrough technologies will emerge, paving the way for a sustainable and hydrogen-powered future. In conclusion, rare-earth-metal-based hydrogen storage materials hold immense potential for revolutionizing the hydrogen storage landscape and enabling the transition towards a sustainable hydrogen economy. With the continued advancements in materials science, characterization techniques, and computational methods, it is anticipated that these materials will play a pivotal role in the development of efficient, safe, and cost-effective hydrogen storage systems. The collaborative efforts of researchers, industry stakeholders, and policymakers will be instrumental in overcoming the existing challenges and realizing the full potential of rare-earth-metal-based hydrogen storage materials in the near future.

## Data Availability

Data sharing is not applicable.
